# Second Reply to Letter to the Editor re: “Clinical utility of the Prostate Health Index (*phi*) for biopsy decision management in a large group urology practice setting”

**DOI:** 10.1038/s41391-019-0166-x

**Published:** 2019-08-28

**Authors:** Jay White, Ronald F. Tutrone, Mark A. Reynolds

**Affiliations:** 1 Carolina Urology Partners, Huntersville, NC USA; 2 Chesapeake Urology Associates, Towson, MD USA; 3 Beckman Coulter, Carlsbad, CA USA; 4 Present Address: The Medical Affairs Company, Kennesaw, GA USA

**Keywords:** Diagnostic markers, Cancer screening

We wish to further respond to the letter of Ehdaie and Carlsson [1], wherein the authors questioned “the lack of any mentioning to the risk of missed high-grade disease” in our originally published article [2]. As stated in the article, our study was designed to address two objectives: (1) to identify the impact of *phi* on decision to biopsy in a community practice setting; and (2) to assess the biopsy yield of cancer with and without the use of *phi*. As reported in our study, we observed a 23.9% reduction in biopsy procedures performed between the Prospective (with *phi*) and Historical (without *phi*) cohorts, along with a modest reduction in the percentage of low-grade (GS 6) cancers detected (9.9% versus 18.4%). However, as reiterated in our first Reply [3], this study was not sufficiently powered to address the question of high-grade vs. low-grade cancers. Simply put, the relatively low numbers of high-grade cancers recorded in our study would not have supported a robust statistical comparison. To illustrate that point, an analysis by Gleason score (GS) is presented below in Table 1.

**Table 1:** Gleason score in historical and prospective cohorts

	Historical (*n* = 683)		Prospective (*n* = 506)		
GS	*n*	Percent (95% Cl)^a^	*n*	Percent (95% Cl)^a^	*p*-value^a^
6	126	18.4 (15.6, 21.6)	50	9.9 (7.3, 12.5)	<0.001
3 + 4	79	11.6 (9.3, 14.2)	31	6.1 (4.0, 8.2)	0.001
4 + 3	24	3.5 (2.3, 5.2)	19	3.8 (2.1, 5.4)	0.807
>7	22	3.2 (2.0, 4.8)	7	1.4 (0.4, 2.4)	<0.001
6 + (3 + 4)	205	30.0 (26.6, 33.6)	81	16.0 (12.8, 19.2)	0.245
(4 + 3) + >7	46	6.7 (4.9, 8.9)	26	5.1 (3.2, 7.1)	0.031

^a^The Wald approach was used to calculate confidence intervals using Proc Freq of SAS, Version 9.4M3 of the SAS System^©^ 2015 (SAS Institute Inc., Cary, NC, USA). All comparisons conducted with *α* = 0.05; *p*-value H_A_: Diff ≠ 0

As shown in the table, the Prospective cohort showed a modest reduction in percentages of GS 6 and GS (3 + 4) cancers detected (differences of 8.5% and 5.5%, respectively), whereas the percentages of GS (4 + 3) cancers were nearly equivalent. The GS > 7 cancers showed overlapping confidence intervals, as one might expect due to the relatively low and variable numbers of high-grade cancers recorded among the four participating sites in our study. Similarly, when pooling the unfavorable intermediate-risk and high-risk patients (GS (4 + 3) and GS > 7), we also find overlapping confidence intervals. Furthermore, when considering just the numbers of positive cancers recorded, we observed an enrichment of high-grade cancers in the Prospective cohort. When utilizing *phi*, our detection rate of clinically significant prostate cancer (GS (4 + 3) and >7) was 24.2% (26/107) versus 18.3% (46/251) for the Historical cohort. These comparisons are offered in the interest of full transparency. Again, the small number of patients with high-grade cancers precludes any formative conclusions.

Nonetheless, the question of high-grade cancer requires further comment. The population of men enrolled in our study had a non-suspicious DRE and a recent total PSA 4-10 ng/mL. Recent guidelines published by the American Urological Association recommend active surveillance be offered to such men with organ-confined prostate cancer, a Gleason Score of ≤(3 + 4) and tPSA <10 ng/mL [4]. Accordingly, our data do not support the assertion offered by Ehdaie and Carlsson that a urologist’s decision to defer biopsy, when guided by clinical judgment along with the *phi* result, presents a significant risk of missing high-grade disease. Furthermore, as reported in our study, patients not biopsied following their initial assessment are expected to be monitored more closely or by additional methodologies. This observation, along with the most recent NCCN guidelines recommending repeat assessment of PSA and DRE within 6–12 months, supports our conclusion that a biopsy was safely deferred in such men.

Finally, we wish to acknowledge that no biomarker test for prostate cancer can fully exclude the risk of missing high-grade disease, and that even the best such tests currently available offer relatively equivalent performance. For example, a recent meta-analysis of 28 studies including 16,762 patients comparing *phi* to 4KScore^®^ concluded that both markers provided comparable diagnostic accuracy in detecting overall and high-grade PCa [5].
